# Perceived cardiovascular disease risk among multi-ethnic postmenopausal women residing in Korea: a Q-methodological study

**DOI:** 10.4069/whn.2026.02.21

**Published:** 2026-03-31

**Authors:** Kyu Ho Lee, Sun Jung Park, Byung Jun Park

**Affiliations:** 1 Kangbuk Samsung Hospital, Seoul, Korea; 2 College of Nursing, Korea University, Seoul, Korea; 3 Department of Nursing, Sahmyook Health University, Seoul, Korea; 4 Department of Nursing, Kangwon National University, Samcheok, Korea

**Keywords:** Cardiovascular diseases, Menopause, Perception

## Abstract

**Purpose:**

This study aimed to explore the subjective perceptions of cardiovascular disease (CVD) risk among multi-ethnic postmenopausal women residing in Korea. Because menopause represents a significant physiological transition that heightens susceptibility to CVD, identifying how these women perceive CVD risk is essential.

**Methods:**

Using Q-methodology, data were collected in November 2024 from 40 participants—Korean, Chinese, and Filipino postmenopausal women residing in Korea who had experienced either natural or surgically induced menopause—who sorted 35 Q-statements on a nine-point scale. The data were analyzed using the PC-QUANL program to identify perception patterns.

**Results:**

Four distinct perception types emerged. Type 1, heightened awareness of CVD, included participants who recognized metabolic and emotional changes after menopause as important risk factors and showed strong vigilance toward prevention. Type 2, passive cardiovascular risk management, included participants who acknowledged some preventive behaviors, such as health checkups, but showed limited engagement in information-seeking or in recognizing menopause-specific risks. Type 3, emphasis on healthy lifestyle, included participants who prioritized dietary control and exercise but paid less attention to physiological changes associated with menopause. Type 4, information-centered self-directed, included participants who actively sought health information but were less likely to translate that knowledge into consistent preventive behaviors.

**Conclusion:**

Postmenopausal women residing in Korea exhibited diverse patterns of awareness and preventive behavior regarding CVD risk. These findings may inform the development of tailored educational and behavioral interventions that address the unique characteristics of each perception type. Furthermore, this study may provide foundational data for designing culturally sensitive CVD prevention programs for multi-ethnic postmenopausal women.

## Introduction

Cardiovascular and cerebrovascular diseases are among the leading chronic conditions and causes of death in South Korea. According to the 2023 Cause of Death Statistics from Statistics Korea [1], the mortality rate from circulatory system diseases was 132.1 per 100,000, with heart disease accounting for 64.8 deaths and cerebrovascular disease accounting for 47.3 deaths per 100,000. Notably, the mortality rate from circulatory system diseases was 1.1 times higher in women (136.3) than in men (127.8), and women had higher mortality rates than men for both hypertensive and cerebrovascular diseases [1]. Numerous studies have examined the high mortality burden of cardiovascular and cerebrovascular diseases in women and have linked it to physical changes and increased fat accumulation. It is well established that the prevalence of cardiovascular and cerebrovascular diseases in women increases significantly after 40 years of age, largely because of hormonal changes related to menopause and aging [2]. In a study conducted to identify risk factors and vulnerable groups for cardiovascular and cerebrovascular diseases among middle-aged women, menopause was identified as a significant factor, along with diabetes, body mass index, and a family history of hypertension [3]. The prevalence of cardiovascular and cerebrovascular diseases is approximately 20% in women older than 50 years, and this rate is 2.6 times higher in women than in men aged 80 years and older [4]. Therefore, improving awareness of disease prevention among postmenopausal women at high risk for cardiovascular and cerebrovascular diseases is essential.

The increased risk of cardiovascular disease (CVD) in postmenopausal women is closely associated with hormonal changes related to menopause. Specifically, declining estrogen levels, accompanied by elevated concentrations of follicle-stimulating hormone and luteinizing hormone, contribute to the development of dyslipidemia [5]. Before menopause, women typically have a more favorable lipid profile than men, characterized by higher levels of high-density lipoprotein and lower levels of low-density lipoprotein. However, the reduction in estrogen after menopause leads to adverse changes in serum lipid and lipoprotein levels, increasing the risk of ischemic heart disease, such as myocardial infarction and angina, as well as cerebrovascular events, including stroke [6]. Sex hormone levels do not remain constant throughout a woman’s lifespan; rather, they begin to decline in the early 40s. This hormonal decline shortens menstrual cycles and ultimately leads to menopause [7]. According to a 2018 survey conducted by the Korea Centers for Disease Control and Prevention, the average age at menopause among Korean women was 49.3 years [8]. The onset of menopause, marked by reduced hormone levels, is associated with a variety of physiological symptoms. The abrupt decline in female hormones during menopause leads to symptoms including vasomotor disturbances, psychological and emotional disorders, skin atrophy, urogenital symptoms, osteoporosis, and atherosclerosis. This reduction in estrogen is particularly important because it diminishes the cardioprotective effect of estrogen, thereby positioning menopause as a recognized risk factor for CVD in women [5]. Moreover, metabolic risk factors relevant to cardiovascular health increase significantly as women transition from premenopause to postmenopause [9]. These risks have been reported to increase fourfold within the first 10 years after natural menopause and to be higher in women than in men [10,11]. Reflecting these findings, the American Heart Association classifies women older than 50 years with one or more cardiovascular risk factors as a high-risk group for CVD [12].

The heightened risk of CVD is an important health concern not only for native Korean women but also for multicultural marriage immigrant women residing in Korea [13]. This issue is further shaped by sociocultural adaptation, as studies indicate that longer residence in Korea is associated with increased selection of Korean-style diets and greater alignment with Korean dietary behavior patterns [14]. Because diet is a significant risk factor for CVD, this shift in eating habits reflects a tangible change in risk exposure over time. Furthermore, prolonged residence in Korea and increased health literacy may lead to broader changes in health-related behaviors in this population [15]. As interest in multicultural health and health management continues to grow, effective programs are urgently needed to improve awareness of CVD prevention and management and to strengthen health behaviors among middle-aged women residing in Korea, including immigrants. As Korea shifts toward a multicultural society, marriage immigrant women and multicultural women have become important constituents of the population, making their health issues essential components of national public health strategies. Exploring subjective perceptions of CVD risk, particularly among women adapting over time to the Korean social environment and dietary patterns, is fundamental to the development of effective, tailored health intervention programs [14]. CVD prevention and management strategies increasingly emphasize active, participant-centered, and often community-based engagement. Research suggests that incorporating small-group dynamics into health behavior promotion programs for middle-aged women can foster enjoyment and belonging, thereby serving as a powerful motivator for behavioral change [16]. Despite this, most existing studies validating the effectiveness of health programs for CVD and metabolic syndrome prevention in postmenopausal women have focused on uniform education and standardized information provision. Given that postmenopausal women are highly vulnerable to metabolic diseases such as CVD, and given the availability of various interventional strategies, a clear gap remains in the literature regarding the subjective investigation of risk perception tailored to the characteristics of this population. In addition, because the number of foreign-born women who have lived long-term in Korea and are now entering menopause is increasing, inclusion of a multi-ethnic population is essential to capture how biological, cultural, and acculturation-related factors shape perceptions of CVD risk. Accordingly, the primary aim of this study was to identify subjective perceptions of CVD risk among postmenopausal women residing in Korea and to systematically categorize these perceptions into thematic domains. This categorization may serve as baseline data for the future development, application, and evaluation of tailored intervention components designed to promote positive health behaviors in this population.

## Methods

**Ethics statement:** This study was approved by the Institutional Review Board of Kangwon National University (No. KWNUIRB-2024-10-003-001). Informed consent was obtained from the participants.

### Study design

**Figure 1.**
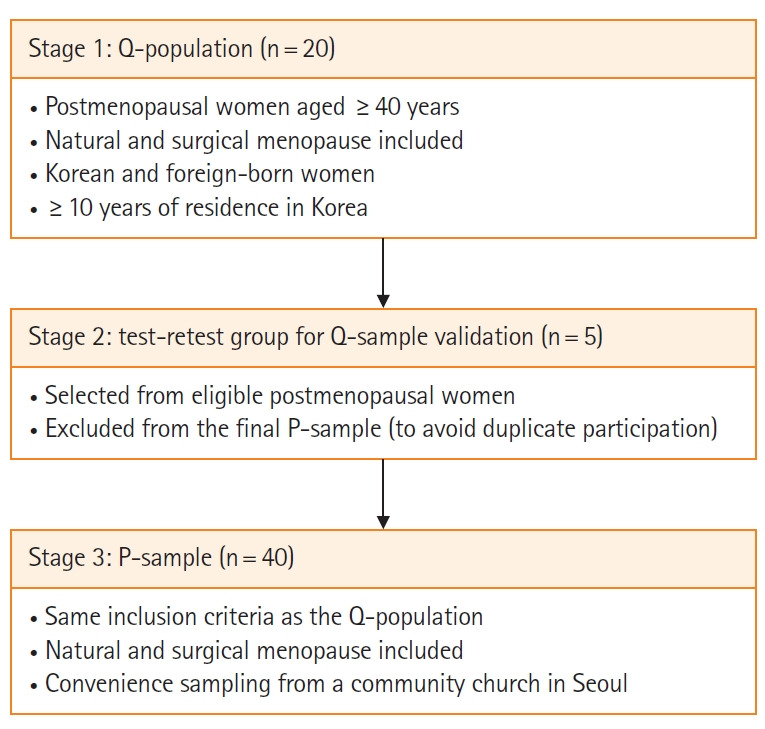
Flow diagram of study procedures and participants.

This study employed Q-methodology to systematically analyze subjective viewpoints. The research design included the development of a Q-sample derived from literature reviews, open-ended questionnaires, and in-depth interviews. This structured design ensured that participants’ subjective perceptions were captured, systematically categorized into typologies, and interpreted with methodological rigor. This study was reported in accordance with the Standards for Reporting Qualitative Research guidelines [17]. A flow diagram of the participants and study procedures is presented in Figure 1.

### Criteria for selecting the Q-population

The Q-population was defined to include women whose experiences were directly relevant to the exploration of subjective perceptions of CVD risk after menopause. Eligibility criteria were as follows: women aged 40 years or older who had experienced natural or surgically induced menopause and were residing in Korea. To reflect sociocultural diversity in health perceptions, both Korean and multicultural women were included. Participants were required to be able to communicate their thoughts and experiences regarding health and disease prevention. Women with severe cognitive impairment or psychiatric conditions that could hinder meaningful participation were excluded. These criteria were established to ensure that the Q-population adequately represented the range of subjective viewpoints relevant to the purpose of this study.

### Construction of the Q-population (concourse)

To ensure that the Q-sample adequately reflected the range of CVD-related perceptions among postmenopausal women, the Q-population was constructed by integrating statements derived from literature reviews, open-ended questionnaires, and individual in-depth interviews. These sources allowed the researchers to capture diverse viewpoints aligned with the aims of the study. In this study, the Q-population, which served as the foundation for development of the Q-sample, was constructed using multiple methods suggested in the literature [18], including a literature review on CVD in postmenopausal women [2,4-6], open-ended questionnaires, and individual in-depth interviews. The literature review involved books related to CVD risk and the characteristics of postmenopausal women, as well as academic journal articles examining their knowledge, attitudes, and perceptions regarding CVD risk. Both Korean and foreign-born women who had resided in Korea for at least 10 years and were postmenopausal, whether through natural or surgical menopause, were eligible for participation. The inclusion of multi-ethnic participants was intentional, as long-term residence in Korea may facilitate acculturation and shape health perceptions, health-seeking behaviors, and adaptation to the Korean healthcare environment. Therefore, their perceptions of CVD risk were considered meaningfully influenced by the Korean sociocultural context, making them appropriate participants for this study. Given that the Q-population in this study consisted of postmenopausal women and that the primary objective was to explore their subjective perceptions and attitudes toward CVD risk, including the diverse perspectives of multicultural postmenopausal women residing in Korea, the following general open-ended questions were developed:

• What is your understanding of CVD based on your current knowledge?

• What do you believe are appropriate treatments and nursing care practices for CVD?

• What strategies do you think are effective for preventing CVD?

• In your opinion, why is CVD considered particularly risky for postmenopausal women?

Trained interpreters were prepared in advance to ensure that non-Korean participants could express their subjective views accurately and comfortably during data collection. From August 1 to August 15, 2024, the researcher and two research assistants visited a church in Seoul to recruit postmenopausal women using convenience sampling, and additional participants were subsequently recruited through snowball sampling. As a result, a total of 20 postmenopausal women of diverse nationalities were included in the Q-population.

### Q-sample construction

Construction of the Q-sample is a critical step in Q-methodology because it involves summarizing and condensing the breadth and depth of content derived from the Q-population. The resulting Q-sample is considered representative of the Q-population and serves as an analytical foundation for the research process [18]. In this study, a panel of four experts was assembled to extract the Q-sample. The panel consisted of the principal investigator, one expert in Q-methodology, one nursing professor specializing in women’s health, and one professor of psychiatric nursing who was also a certified mental health nurse with expertise in subjective perception. The panel repeatedly reviewed the statements over three rounds of discussion, during which redundant and ambiguously worded statements were eliminated and similar items were consolidated. As a result, a final set of 35 statements was selected to constitute the Q-sample, consistent with the general recommendation that Q-samples range from 20 to 100 statements [19].

### Validity and reliability verification of the Q-sample

To evaluate the validity of the Q-sample, content validity was established through a comprehensive review of the literature and previous studies on CVD in postmenopausal women conducted by the researcher, as well as through expert evaluation of the appropriateness of the 35 selected statements by a panel of four specialists. Face validity was further confirmed through in-person meetings with the expert panel, during which the statements were revised and refined for clarity and relevance. To assess the reliability of Q-sorting, a test-retest procedure was conducted using the finalized set of 35 statements. Five Korean postmenopausal women, who voluntarily agreed to participate in the reliability verification process and were not included in the Q-population, completed the Q-sorting individually in person. One week later, they performed the same sorting task again under identical conditions. During this process, the appropriateness of wording and phrasing was also reviewed. The resulting Pearson correlation coefficient exceeded .80, indicating satisfactory reliability of the Q-sample.

### P-sample selection

Both Korean and foreign-born postmenopausal women who had resided in Korea for at least 10 years were eligible for participation. To facilitate accurate communication during the Q-sorting and interview processes, trained interpreters assisted non-Korean participants as needed; therefore, these participants were also included in the P-sample.

The P-sample was selected using the same inclusion criteria as the Q-population. Eligible participants were postmenopausal women of Korean, Filipino, or Chinese nationality who had lived in Korea for at least 10 years, were able to understand the study procedures either in Korean or with the assistance of an interpreter and voluntarily agreed to participate.

Because the task of the P-sample was to sort the Q-sample statements into a forced distribution, and although Q-methodology allows flexibility in sample size, an excessively large P-sample may dilute the interpretive clarity of each typology by loading too many participants onto a single factor. In accordance with small-sample theory, which emphasizes the exploration of diverse subjective viewpoints rather than statistical representativeness and considering the general recommendation that the P-sample remain at approximately 40 to 50 participants or fewer [20], 40 postmenopausal women were recruited for this study (Table 1).

Participants were recruited using convenience sampling from a large church located in Seoul. No individual participated more than once, and duplicate participation was not permitted.

### Q-sorting and formation of Q types

The Q-sorting procedure was conducted from November 1 to November 30, 2024. Participants sorted the finalized 35 Q-sample statements into a forced distribution using a Q-sort distribution grid. A 9-point scale ranging from −4 (strongest disagreement) to +4 (strongest agreement) was applied, which is appropriate for studies using fewer than 40 statements [19]. Participants first classified the statements into three categories—agree, neutral, and disagree—and then placed them on the forced-distribution grid. The final distribution followed a quasi-normal pattern. To facilitate interpretation of the Q types, the two statements placed at the extreme ends of the distribution (+4 and −4) were regarded as representing each participant’s most salient viewpoints [21]. Participants provided written explanations for their selection of these extreme statements. Each Q-sorting session took approximately 30 minutes.

The completed Q-sorts were analyzed using factor analysis to identify shared patterns of subjectivity, and Q types were derived on the basis of these patterns. Four Q types were identified, accounting for a total of 48.3% of the variance. Type 1 explained 27.6% of the variance, followed by type 2 (8.7%), type 3 (6.4%), and type 4 (5.6%) (Table 2).

Intercorrelations among the four Q types ranged from r=−0.05 to 0.67 (Table 3), indicating moderate relationships. These findings suggest that the Q types were relatively independent and represented distinct patterns of perception.

### Data analysis

Data were analyzed using PC-QUANL, a software package designed for Q-methodological research. The 35 statements sorted on the Q-distribution grid were first converted into standardized scores and coded accordingly. Principal component factor analysis with Varimax rotation was then conducted using PC-QUANL. During factor extraction, factors with eigenvalues greater than 1.0 were retained, resulting in the identification of four distinct types. The statements loading on each factor were expressed as standardized scores (Z-scores), and only those with scores of 1.0 or higher were considered significant for interpretation.

## Results

### General characteristics of the P-sample

Of the 40 participants, most were Korean (n=31, 77.5%), and 24 participants (60.0%) were aged 50 years or older. Menstrual cessation (complete menopause) occurred before age 40 years in five participants (12.5%), during their 40s in 27 participants (67.5%), and after age 50 years in eight participants (20.0%). More than two-thirds of participants (n=27, 67.5%) rated their health as average or better. However, when comparing their current health with that of 1 year earlier (n=37, 92.5%) or with that of their peers (n=33, 82.5%), most reported their health as average or worse. Twenty-five participants (62.5%) reported having one or more medical conditions, the most common of which was hypertension (n=10), followed by diabetes (n=7), joint disorders (n=3), and other conditions (n=3; thyroid disease and respiratory disease).

### Characteristics of the types

The distribution of participants across the four types showed that 15 individuals belonged to type 1, seven to type 2, 10 to type 3, and eight to type 4. The demographic characteristics and factor weights of participants in each type are presented in the Supplementary Table 1. Within each type, participants with higher factor weights were considered to exhibit the most prototypical characteristics of that type and thus served as representative cases. To analyze subjective perceptions of CVD risk among postmenopausal women residing in Korea by type, the interpretation focused on statements showing strong agreement (Z-score ≥+1) and strong disagreement (Z-score ≤−1) among the 35 Q-sample statements. The characteristics of each type were identified on the basis of statements for which the standardized scores of a specific type differed markedly from those of the other types. Based on these analytical criteria, the typologies of postmenopausal women’s perceptions regarding CVD risk are presented below (Table 4).

Participants with high factor loadings (≥0.8) on a specific factor were regarded as representative cases of that Q type. As such, particular attention was given to their responses, demographic characteristics, and perspectives, as they served as key exemplars of the typology. This principle was applied in the present study to classify postmenopausal women’s perceptions of CVD risk. Accordingly, four distinct Q types were identified, each represented by participants with the highest factor loadings within their respective groups (Table 4).

#### Type 1: heightened awareness of cardiovascular disease 

Participants classified under type 1 showed the highest level of agreement with the statements “After menopause, a slower metabolism increases the likelihood of weight gain” (Z=1.81), “Physical and emotional stress resulting from menopause negatively affects cardiovascular health” (Z=1.79), and “After menopause, increased blood pressure and cholesterol levels may raise the risk of CVD” (Z=1.49). In contrast, type 1 participants showed the strongest disagreement with the statements “I consistently take medication as prescribed by my physician as prescribed by my physician” (Z=–2.00), “I try to avoid spicy and hot foods” (Z=–1.65), and “I openly express emotional and cognitive discomfort, such as anxiety, depression, or forgetfulness” (Z=–1.60). These results suggest that postmenopausal women classified under type 1 were aware of symptoms associated with metabolic changes after menopause and recognized the effects of both physical and psychological issues on cardiovascular health. They perceived cardiovascular health as an important concern after menopause and demonstrated a strong tendency toward preventive health behaviors. Accordingly, this group was labeled the “heightened awareness of CVD type.”

#### Type 2: passive cardiovascular risk management

Participants in type 2 showed the highest level of agreement with the statements “I believe that regular health checkups are necessary for the early detection and prevention of CVD” (Z=2.26), “I recognize that hypertension is a major risk factor for CVD and regularly monitor my blood pressure” (Z=1.74), and “I am aware that quitting smoking and reducing alcohol consumption are essential for the prevention of CVD” (Z=1.56). Conversely, type 2 participants showed the strongest disagreement with the statements “I obtain information related to CVD through the internet, health magazines, or medical institutions” (Z=–1.72), “Menopausal women consult doctors about their cardiovascular health” (Z=–1.52), and “It is recognized that CVD poses a heightened risk for menopausal women” (Z=–1.36). These findings suggest that participants in type 2 recognized the importance of regular health checkups but showed relatively low engagement in consulting physicians or acknowledging CVD as a major health concern. Although they perceived CVD as a health issue, their intention to engage in preventive measures appeared comparatively low. Therefore, this group was labeled the “passive cardiovascular risk management type.”

#### Type 3: emphasis on healthy lifestyle

Participants in type 3 placed strong emphasis on the role of healthy habits in preventing CVD. They most strongly agreed with the statement “I believe that a diet rich in vegetables, fruits, fiber, and adequate protein intake is important” (Z=2.09). This was followed by agreement with the statements “I tend to reduce the intake of saturated fats and trans fats” (Z=1.97) and “Menopausal women recognize the importance of healthy lifestyle habits in preventing CVD” (Z=1.70). Conversely, type 3 participants showed the strongest disagreement with the statements “Estrogen levels drop sharply during menopause” (Z=–2.32), “I openly express emotional and cognitive discomfort, such as anxiety, depression, or forgetfulness” (Z=–1.52), and “After menopause, increased blood pressure and cholesterol levels may raise the risk of CVD” (Z=–1.48). These findings indicate that participants in type 3 showed strong interest in diet and healthy lifestyle practices and had a clear tendency to engage in behaviors such as dietary control and regular exercise to maintain cardiovascular health. Accordingly, this group was designated the “emphasis on healthy lifestyle type.”

#### Type 4: information-centered self-directed

Participants classified under type 4 expressed a strong interest in acquiring accurate information related to cardiovascular health. They showed the highest level of agreement with the statement “I actively seek information about risk factors and prevention methods for CVD” (Z=1.91). This was followed by strong agreement with “I try to manage stress effectively” (Z=1.75) and “I obtain information related to CVD through the internet, health magazines, or medical institutions” (Z=1.56). Conversely, type 4 participants showed the strongest disagreement with the statements “I consistently take medication as prescribed by my physician” (Z=–1.58), “I am aware that quitting smoking and reducing alcohol consumption are essential for the prevention of CVD” (Z=–1.40), and “I recognize that regular aerobic exercise is beneficial for cardiovascular health” (Z=–1.35). These findings suggest that participants in type 4 placed strong emphasis on identifying preventive factors and actively acquiring information through sources such as the internet. They appeared highly interested in obtaining accurate information and were motivated to remain informed as a means of preventing CVD. Accordingly, this group was designated the “information-centered self-directed type.”

### Consensus items across types

Based on the above results, subjective perceptions of CVD risk among postmenopausal women residing in Korea can be classified into four distinct types, each demonstrating clear and distinguishable characteristics. However, certain statements regarding CVD risk were commonly agreed upon or disagreed upon across all four types, as presented in Table 5. The statements showing overall disagreement included the following: “I believe that menopause affects the risk of CVD” and “I am aware that hormonal changes after menopause can affect CVD risk.” These findings suggest that although postmenopausal women may acknowledge the general importance of CVD risk and its management, they may lack awareness of the direct effects of menopause on cardiovascular health.

## Discussion

The health status findings in this study showed that although 67.5% of participants rated their subjective health as average or better, more than half perceived their current health as average or worse when compared with their health 1 year earlier or with that of their peers. Furthermore, 62.5% of participants had chronic diseases such as hypertension, diabetes, and joint disorders. Collectively, these findings underscore the need for education on CVD prevention and management for postmenopausal women.

The first typology, heightened awareness of CVD type, demonstrated strong recognition of risk factors such as metabolic decline, stress, and elevated blood pressure or cholesterol after menopause, yet participants in this type were less likely to adhere to medication regimens, follow dietary restrictions, or express emotional distress. This pattern is consistent with previous findings indicating that high disease awareness does not always translate into health-promoting behaviors because of personal and social barriers [22,23]. Specifically, prior research on postmenopausal women has reported that despite high disease awareness, various individual and social barriers may prevent the transition to health-promoting actions [22]. In particular, physical changes and emotional instability associated with menopause have been cited as major factors inhibiting health behaviors [24]. Therefore, for women categorized as the heightened awareness type, strategies should go beyond maintaining and reinforcing awareness and should include specific, systematic interventions aimed at promoting health behavior implementation. Developing tailored educational programs for CVD prevention, together with emotional support and self-efficacy enhancement programs, may help bridge the knowledge-to-action gap.

The passive cardiovascular risk management type had basic preventive knowledge but showed limited information-seeking and healthcare engagement, reflecting low perceived susceptibility [25,26]. This finding is similar to previous reports showing that middle-aged women often exhibit insufficient preventive behaviors because, despite awareness of CVD, their perception of personal risk or disease severity remains low [27]. Furthermore, although postmenopausal women face increased CVD risk because of biological changes, lack of access to relevant information and limited communication with healthcare professionals often prevent implementation of preventive measures [7]. Consequently, for the passive cardiovascular risk management type, education that clearly communicates the seriousness of CVD and its association with menopause should be prioritized. In addition, program development should facilitate consultation with healthcare providers alongside active information provision, and motivational strategies should be offered concurrently to support the transition to preventive health behaviors.

The third typology, emphasis on healthy lifestyle type, actively practiced dietary and physical activity behaviors but paid less attention to physiological or emotional changes associated with menopause [26]. This pattern is consistent with previous research suggesting that although middle-aged women recognize the importance of practical habit changes for CVD prevention, their understanding and acceptance of menopause-related biological changes or emotional issues may be relatively limited [28]. Accordingly, it is necessary to maintain the emphasis on a healthy lifestyle for this type while simultaneously providing education on postmenopausal physiological changes and emotional support programs to promote integrated physical and psychological management.

The information-centered self-directed type proactively sought information but showed limited translation of that knowledge into behavior, a pattern consistent with existing studies highlighting the gap that may exist between knowledge acquisition and health behavior implementation [24,29]. Therefore, for this type, intervention strategies should extend beyond simple information provision and instead focus on behavior-change approaches that help participants translate acquired knowledge into practice. Specifically, programs should be developed to enhance information credibility and promote implementation through motivational support and goal-setting, thereby reinforcing the intention to act. In addition, because this study employed a cross-sectional design, longitudinal research is needed to better understand changes in perception and behavior over time and to evaluate the sustained effects of tailored interventions.

Because the sample included both women who experienced natural menopause and those who underwent surgical menopause, the findings reflect a heterogeneous group of postmenopausal women with varied menopausal experiences. However, the relative proportions of natural and surgical menopause were not systematically documented, which limits the ability to examine potential differences between these groups. Given that surgical menopause is associated with abrupt estrogen loss and may intensify menopausal symptoms or influence awareness of CVD differently, this issue should be considered when interpreting the perception patterns identified in this study. In addition, although Q-methodology is effective for capturing subjective viewpoints, its forced-distribution format may restrict participants’ ability to fully express their priorities [17]. In this study, approximately two-thirds of the participants were younger than 55 years. This age distribution may limit the generalizability of the findings to older postmenopausal women, who are at higher risk for CVD and may hold different perceptions. However, this characteristic is also a strength of the study, as it provides valuable insight into CVD awareness among relatively younger postmenopausal women, a group in which early recognition and preventive attitudes are particularly important.

The findings of this study have several implications for women’s health nursing, clinical practice, and public health programs targeting multi-ethnic postmenopausal women in Korea. First, the four distinct perception types highlight the need for tailored CVD prevention strategies rather than uniform educational approaches. Women in type 1, who showed high awareness but inconsistent behavioral execution, may require behavior-focused interventions such as structured coaching, self-monitoring tools, and counseling to strengthen self-efficacy and support sustained lifestyle modification. Women in type 2 demonstrated low perceived susceptibility and limited engagement with healthcare services, suggesting the need for enhanced risk communication, culturally sensitive education, and facilitated access to medical consultations, particularly for immigrant women who may face linguistic or systemic barriers. Women in type 3 prioritized healthy routines yet lacked physiological and emotional understanding of menopause, indicating the need for integrated education on hormonal changes, menopausal symptoms, stress management, and emotional regulation to promote a more comprehensive approach to CVD prevention. Women in type 4, characterized by active information-seeking but limited behavioral adherence, may benefit from individualized goal-setting, digital reminders, follow-up consultations, and nurse-led behavioral guidance to ensure that increased knowledge is translated into consistent action.

Across all types, this study underscores the importance of culturally responsive and context-specific nursing interventions, especially given that many participants were immigrant women navigating the dual challenges of menopause and persistent adaptation-related issues even after cultural adjustment [30]. Multilingual resources and community-based health support may help reduce the perception–behavior gap identified in this study. Furthermore, the typological insights provide a foundational framework for developing precision health strategies aligned with each group’s beliefs, motivations, and barriers, which may ultimately contribute to more effective CVD prevention among postmenopausal women living in Korea.[Table t1-whn-2026-02-21]

## Figures and Tables

**Table 1. t1-whn-2026-02-21:** General characteristics of participants (N=40)

Variables	Categories	n (%)
Nationality	Korean	31 (77.5)
	Filipino	4 (10.0)
	Chinese	5 (12.5)
Age (year)	40–45	5 (12.5)
	46–50	11 (27.5)
	51–55	11 (27.5)
	≥55	13 (32.5)
Age of menstrual cessation (year)	≤40	5 (12.5)
	41–45	16 (40.0)
	46–50	11 (27.5)
	51–55	8 (20.0)
Religion	Christian	14 (35.0)
	Buddhist	12 (30.0)
	Catholic	4 (10.0)
	None	10 (25.0)
Self-rated health status	Very poor	2 (5.0)
	Poor	11 (27.5)
	Fair	16 (40.0)
	Good	10 (25.0)
	Very good	1 (2.5)
Health status compared with 1 year ago	Very poor	1 (2.5)
	Poor	21 (52.5)
	Fair	15 (37.5)
	Good	3 (7.5)
	Very good	0 (0)
Health status compared with others	Very poor	2 (5.0)
	Poor	11 (27.5)
	Fair	20 (50.0)
	Good	7 (17.5)
	Very good	0 (0)
Current disease status	No	15 (37.5)
	Yes	25 (62.5)
	Type of diseases	
	Hypertension	10 (25.0)
	Diabetes	7 (17.5)
	Joint disorders	3 (7.5)
	Others	3 (7.5)
	Cerebrovascular disease	1 (2.5)
	Gastrointestinal disease	1 (2.5)

**Table 2.** Eigenvalues, variance, and cumulative percentages (N=40)

Variables	Type 1	Type 2	Type 3	Type 4
Eigenvalue	11.02	3.49	2.55	2.26
Variance (%)	27.6	8.7	6.4	5.6
Cumulative variance	0.27	0.36	0.42	0.48

Type 1: Heightened awareness of CVD type (n=15); Type 2: passive cardiovascular risk management type (n=7); Type 3: emphasis on healthy lifestyle type (n=10); Type 4: information-centered self-directed type (n=8).

**Table 3.** Correlation matrix between types (N=40)

Variables	Type 1	Type 2	Type 3	Type 4
Type 1	1.00			
Type 2	0.21	1.00		
Type 3	0.67	0.34	1.00	
Type 4	–0.31	–0.05	–0.12	1.00

Type 1: Heightened awareness of CVD type (n=15); Type 2: passive cardiovascular risk management type (n=7); Type 3: emphasis on healthy lifestyle type (n=10); Type 4: information-centered self-directed type (n=8).

**Table 4.** Representative Q-samples and Z-scores in types (N=40)

Statements	Z-score
Type 1. Heightened awareness of CVD type (n=15)	
3. After menopause, a slower metabolism increases the likelihood of weight gain	1.81
4. Physical and emotional stress resulting from menopause negatively affects cardiovascular health	1.79
2. After menopause, increased blood pressure and cholesterol levels may raise the risk of CVD	1.49
5. I try to maintain healthy lifestyle habits to prevent CVD after menopause	1.44
6. I am concerned that hormone replacement therapy may increase the risk of CVD	1.25
29. I try to consume sufficient amounts of calcium, and vitamins E, B, and D	–1.00
30. I try to avoid wearing tight-fitting clothing such as skinny jeans, pantyhose, and body-shaping undergarments	–1.03
33. I try to get at least 7 hours of sufficient sleep each day	–1.54
31. I openly express emotional and cognitive discomfort, such as anxiety, depression, or forgetfulness	–1.60
32. I try to avoid spicy and hot foods	–1.65
34. I consistently take medication as prescribed by my physician	–2.00
Type 2. Passive cardiovascular risk management type (n=7)	
15. I believe that regular health checkups are necessary for the early detection and prevention of CVD	2.26
16. I recognize that hypertension is a major risk factor for CVD and regularly monitor my blood pressure	1.74
14. I am aware that quitting smoking and reducing alcohol consumption are essential for the prevention of CVD	1.56
31. I openly express emotional and cognitive discomfort, such as anxiety, depression, or forgetfulness	1.18
27. I am aware that hormonal changes after menopause can affect CVD risk	–1.01
23. I seek CVD prevention strategies that are tailored to women	–1.15
19. I recognize the importance of stress management	–1.20
24. I am aware of the major causes of CVD	–1.23
22. I actively seek information about risk factors and prevention methods for CVD	–1.25
20. I believe that practices such as yoga, meditation, and deep-breathing exercises help maintain cardiovascular health	–1.29
26. It is recognized that CVD poses a heightened risk for menopausal women	–1.36
7. Menopausal women consult doctors about their cardiovascular health	–1.52
21. I obtain information related to CVD through the internet, health magazines, or medical institutions	–1.72
Type 3. Emphasis on healthy lifestyle type (n=10)	
11. I believe that a diet rich in vegetables, fruits, fiber, and adequate protein intake is important	2.09
12. I tend to reduce the intake of saturated fats and trans fats	1.97
10. Menopausal women recognize the importance of healthy lifestyle habits in preventing CVD	1.70
35. I try to manage stress effectively	1.43
9. Menopausal women recognize that CVD is a major health concern	1.22
5. I try to maintain healthy lifestyle habits to prevent CVD after menopause	1.19
30. I try to avoid wearing tight-fitting clothing such as skinny jeans, pantyhose, and body-shaping undergarments	–1.10
32. I try to avoid spicy and hot foods	–1.32
2. After menopause, increased blood pressure and cholesterol levels may raise the risk of CVD	–1.48
31. I openly express emotional and cognitive discomfort, such as anxiety, depression, or forgetfulness	–1.52
1. Estrogen levels drop sharply during menopause	–2.32
Type 4. Information-centered self-directed type (n=8)	
22. I actively seek information about risk factors and prevention methods for CVD	1.91
35. I try to manage stress effectively	1.75
21. I obtain information related to CVD through the internet, health magazines, or medical institutions	1.56
23. I seek CVD prevention strategies that are tailored to women	1.51
5. I try to maintain healthy lifestyle habits to prevent CVD after menopause	1.35
24. I am aware of the major causes of CVD	1.26
20. I believe that practices such as yoga, meditation, and deep-breathing exercises help maintain cardiovascular health	1.00
32. I try to avoid spicy and hot foods	–1.01
16. I recognize that hypertension is a major risk factor for CVD and regularly monitor my blood pressure	–1.02
17. I monitor my blood cholesterol levels and try to maintain a balance between LDL and HDL cholesterol	–1.08
31. I openly express emotional and cognitive discomfort, such as anxiety, depression, or forgetfulness	–1.16
33. I try to get at least 7 hours of sufficient sleep each day	–1.21
13. I recognize that regular aerobic exercise is beneficial for cardiovascular health	–1.35
14. I am aware that quitting smoking and reducing alcohol consumption are essential for the prevention of CVD	–1.40
34. I consistently take medication as prescribed by my physician	–1.58

CVD: Cardiovascular disease; HDL: high-density lipoprotein; LDL: low-density lipoprotein.

**Table 5.** Consensus items and average Z-scores (N=40)

Q-statements	Z-scores
Q25. I believe that menopause affects the risk of CVD	–0.44
Q27. I am aware that hormonal changes after menopause can affect CVD risk	–0.70

CVD: Cardiovascular disease.

## Supplementary materials

Further details on supplementary materials are presented online (available at https://doi.org/10.4069/whn.2026.02.21).

Supplementary Table 1. Demographic characteristics and factor weights for P-sample (N=40)
