# Supplementary material for: Perceived cardiovascular disease risk among multi-ethnic postmenopausal women residing in Korea: a Q-methodological study
Source: Womens Health Nurs. 2026 Mar 31;32(1):40–51. doi: 10.4069/whn.2026.02.21 (PMC13071678; doi:10.4069/whn.2026.02.21)
Supplement: Supplementary Table 1. — Demographic characteristics and factor weights for P-sample (N=40) [file whn-2026-02-21-Supplementary-Table-1.pdf]

Supplementary Table 1. Demographic characteristics and factor weights for P-sample (N=40)

Type	Variable no.	Factor weight	Nationality	Age	Age of menstrual cessation	Medical condition	No. of medical condition
Type 1 (n = 15)	3	4.29	Korean	50s	40s	Yes	Hypertension
	4	3.13	Korean	40s	40s	Yes	Joint disorders
	13	0.34	Korean	50s	40s	Yes	Hypertension
	15	2.16	Korean	50s	30s	Yes	Hypertension
	16	2.74	Korean	40s	40s	Yes	Others
	17	1.83	Korean	40s	40s	No	-
	24	1.63	Korean	50s	40s	No	-
	25	0.7	Chinese	50s	30s	Yes	Others
	28	1.04	Chinese	50s	40s	No	-
	29	0.74	Chinese	50s	40s	Yes	Joint disorders
	31	1.75	Filipino	50s	40s	No	-
	32	0.75	Korean	50s	50s	Yes	Hypertension
	36	1.43	Korean	40s	40s	No	-
	39	0.78	Korean	40s	40s	No	-
	40	1.45	Korean	40s	40s	No	-
Type 2 (n = 7)	5	0.23	Filipino	50s	40s	No	-
	8	0.59	Korean	50s	40s	Yes	Hypertension
	9	0.91	Korean	50s	30s	Yes	Diabetes
	19	0.69	Korean	50s	50s	Yes	Diabetes
	20	0.63	Chinese	50s	50s	Yes	Diabetes
	33	0.96	Korean	40s	40s	No	-
	37	0.18	Korean	50s	50s	Yes	Cerebrovascular disease
Type 3 (n = 10)	1	0.23	Filipino	50s	40s	No	-
	2	0.91	Korean	40s	40s	No	-
	10	0.44	Korean	50s	30s	Yes	Diabetes
	18	0.51	Korean	50s	50s	No	-
	23	0.62	Korean	50s	40s	Yes	Gastrointestinal disease
	26	0.72	Korean	40s	40s	No	-
	27	0.45	Chinese	50s	30s	Yes	Others
	30	0.09	Korean	50s	40s	Yes	Joint disorders
	35	1.21	Korean	40s	40s	No	-
	38	1.1	Korean	40s	40s	Yes	Hypertension
Type 4 (n = 8)	6	1.46	Filipino	50s	50s	Yes	Hypertension
	7	0.68	Korean	50s	50s	Yes	Hypertension
	11	0.5	Korean	50s	40s	Yes	Hypertension
	12	0.35	Korean	40s	30s	Yes	Diabetes
	14	0.75	Korean	40s	40s	Yes	Diabetes
	21	1.01	Korean	50s	50s	Yes	Hypertension
	22	0.69	Korean	40s	40s	Yes	Diabetes
	34	1.73	Korean	40s	40s	No	-

Type 1: heightened awareness of CVD type (n=15); Type 2: passive cardiovascular risk management type (n=7); Type 3: emphasis on healthy lifestyle type (n=10); Type 4: information-centered self-directed type (n=8).
